# Development and Application of the Quality Index Method for Ice-Stored King Weakfish (*Macrodon ancylodon*)

**DOI:** 10.3390/foods13172844

**Published:** 2024-09-07

**Authors:** Rafaela Cristina Barata Alves, Enrique Pino-Hernández, Jhonatas Rodrigues Barbosa, Elen Vanessa Costa da Silva, Consuelo Lúcia Sousa de Lima, Raul Coimbra Miranda, Lúcia de Fátima Henriques Lourenço

**Affiliations:** 1 LAPOA/FEA (Faculty of Food Engineering), Federal University of Pará (UFPA), Belém 66075-110, PA, Brazil; rafaela.balves@gmail.com (R.C.B.A.); sousa@ufpa.br (C.L.S.d.L.); raul.miranda@icb.ufpa.br (R.C.M.); 2 Institute of Animal Health and Production—ISPA, Federal Rural University of Amazonia (UFRA), Belém 66077-830, PA, Brazil; jhonquimbarbosa@gmail.com; 3 INOV.LINEA/TAGUSVALLEY—Science and Technology Park, 2200-062 Abrantes, Portugal; enrique.hernandez@tagusvalley.pt; 4 Food Technology Department, Pará State University (UEPA), Belém 66050-540, PA, Brazil; elen.vanessa@uepa.br

**Keywords:** QIM, sensory evaluation, shelf-life

## Abstract

The freshness of raw fish has become one of the industry’s and consumers’ main concerns regarding quality, safety, and shelf-life estimation. To determine the freshness of the king weakfish (*Macrodom ancylodom*), the quality index method (QIM) was employed for sensory analyses, along with the assessment of proximate composition, pH, total volatile bases (TVB-N), thiobarbituric acid reactive substances (TBARS), biogenic amines, fatty acids, texture, and microbiological parameters. The results show that the QIM obtained over the storage period exhibited a linear increase, ranging from 2 to 21 demerit points, with a high correlation (R^2^ = 0.9868) among the data. The microbiological results indicated an increase in the counts of psychrotrophic and mesophilic bacteria throughout the storage period. TVB-N values ranged from 11 to 28 mg/100 g, and TBARS values ranged from 0.235 to 0.298 mg MDA/kg when stored in ice. The presence of putrescine, cadaverine, spermidine, and toxic volatile compounds was a potential indicator of fish freshness. Based on the correlation between the methods considered indicators of freshness and quality, it can be concluded that the king weakfish maintains its commercial stability for up to 11 days when stored in ice.

## 1. Introduction

The quality index method (QIM) is used to assess fish freshness, widely acknowledged as the most efficient sensory classification method for reliably establishing quality due to its minimal training requirements, accuracy, and non-destructive nature. The QIM differs from other sensory approaches, such as the Torry scale, which rely on standardized attributes for groups of organisms, to accommodate variations in perception among aquatic organisms [1].

The QIM is a sensory procedure validated by physical, chemical, and microbiological methods and performed by trained food tasters. The protocol QIM is a list of attributes scored in demerits, where the sum of such demerits produces the quality index (QI). The method predicts the equivalent day of storage, the remaining shelf-life for each species, and the rejection time (spoiled) for consumption. Furthermore, the QIM has shown satisfactory results in the fishing industry when evaluating changes in quality attributes such as appearance, eye, gills, and abdomen [2].

Because of the complexity of chemical/biochemical and quality parameters in fish, alternative methods are highly required to evaluate shelf-life of food products based on the correlation of distinct quality criteria [3]. Volatile organic substances are chemicals used to check the quality of fish. They are closely related to species-specific traits, such as habitat, diet, and storage conditions. The lack of information regarding the concentration of biogenic compounds and other nutritional aspects (for example, the profile of fatty acids) makes it necessary to evaluate these quality indicators for each fish species during commercial storage periods [4].

Different fish species have already been tested using the QIM, for example, *Scomber japonicus* [5], *Boops boops* L. [6], *Panullirus argus* [7], *Oncorhynchus mykiss* [8], *Oreochromis niloticus* [1], and farmed ones such as *Sparus aurata, Dicentrarchus labrax* [9], and *Colossoma Macropomum* [10]. King weakfish (*Macrodon ancylodon*) is a species of fish that is important in Brazil, especially on the north and northeast coasts. It is found in large numbers during the harvest period and is very popular in local markets. In Brazil, the consumption of fish by the Brazilian population is, on average, approximately 9 kg/inhabitant/year. The Food and Agriculture Organization’s (FAO’s) recommendation is to consume 12 kg per person per year. According to data from the Ministry of Agriculture and Livestock of the Brazilian government, the consumption of fish in the Amazon hydrographic region is close to 150 kg per person per year.

Brazil stands out for being a major producer of fish, in addition to having a diversity of freshwater and saltwater aquatic species. Fish is a part of the Brazilian cultural diversity and is used in many dishes. The growth of exports and continuous investment of companies in new products requires the development of quality and food safety protocols. The objective was to determine the freshness of king weakfish stored in ice through sensory analysis (QIM protocol) and microbiological, physicochemical, and biochemical markers.

## 2. Materials and Methods

### 2.1. Sample Obtention and Storage

**Figure 1:**
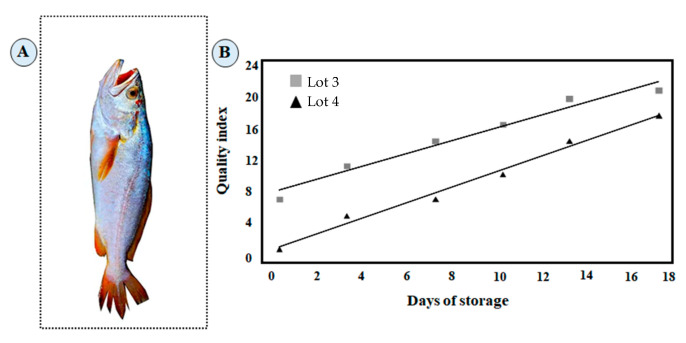
King weakfish (*Macrodon acylodon*) (**A**) and linear correlation between days of storage in ice and quality index for whole king weakfish (**B**).

A total of 95 specimens of king weakfish (mean weight of 460 g and mean length of 32 cm) were collected during the rainy season in the mouths of estuaries in the region of the Pará River, 150 km apart from the Marajó coast (Figure 1A). The fish were divided into four lots obtained during the summer months of July (lot 1), August (lot 2), and September (lot 3), and in the winter month of February (lot 4). The samples were stored in plastic boxes while covered in ice at a 1:1 ratio. A thin plastic film was used to prevent direct contact with the ice. The boxes were kept in refrigerated storage (1 ± 0.5 °C) for 18 days, and ice was replenished daily to keep the fish at 0 °C. The QI for whole king weakfish at 0 days of storage is shown in Figure 1B.

To evaluate the quality parameters, five fish specimens per day from each lot were analyzed. Three whole fish were used in the sensory and physical chemical analysis, and two for microbiological studies.

The sensory analysis was carried out at the fishing industry facilities, while the other studies were performed at the Laboratory of Products of Animal Origin of the Federal University of Pará.

The nature of the activities planned for the sensory analysis was limited to applying already-established scientific and technological knowledge to improve the industrial processes of a Brazilian fish processing company, aiming to facilitate the exchange of expertise between the university and the partner company. Therefore, the tasters were company employees who evaluated only visual characteristics such as general appearance, eyes, gills, and muscle texture. 

### 2.2. Development of QIM

The methodology used to develop the QIM protocol for whole king weakfish stored in ice was based on the protocol previously developed for Acoupa weakfish (*Cynoscion acoupa*) fish [8]. Lot 1 was used to obtain the preliminary protocol (Phase 1); lot 2, to train the panel members (Phase 2); and lots 3 and 4, to apply the QIM (Phase 3). Referee selection and QIM protocol development took place at the fish industry and four referees (one man and three women) were selected among the quality control employees. Training for the QIM began with a session clarifying the method’s foundations and principles. Each session lasted for about 1 h and took place in a properly illuminated room at 20 °C while the referees wore masks, gloves, and hoods. The samples were obtained at random, removed from ice 30 min before the beginning of each sensorial session, and kept in light-colored trays, identified by 3-digit numbers, without information about fish storage for the referees.

In Phase 1, the sensory changes every 24 h in whole king weakfish samples stored in ice were written down by the referees on assessment cards using the main sensory parameters observed by the referees to obtain the preliminary QIM.

In Phase 2, the referees were trained during the assessment of the sample with no knowledge of the day of storage. A final version of the QIM for whole king weakfish stored in ice was drafted from the consensus based on the final comments and suggestions by the referees.

The QI was obtained in the last phase by applying the final QIM developed during the shelf-life assay using lots 3 and 4. Fish sampling was performed on days 1, 4, 8, 11, 14, and 18, with the referees being blind to the storage day.

### 2.3. Physical Chemical Analysis and Instrumental Texture

Moisture, proteins, lipids, ashes, and pH were analyzed only in the fish muscle following the methods previously used in pirarucu fish (*Arapaima gigas*) [11,12]. Centesimal composition was performed only at the initial and final point of the study, since changes in composition are irrelevant during storage period as previously reported [2,4].

Instrumental texture was analyzed in triplicate using a QTS-25 texture analyser (Brookfield CNS, United Kingdom)through the methodology in [11] and using the following test conditions: room temperature, compression force measurement, test speed of 2.0 mm/s, trigger point of 0.1 N, distance 214 mm, target value 10 N, and cylindrical probe (36 mm). Samples were cut into 2 × 2 × 1 cm cubes to determine firmness (consistency).

### 2.4. Total Volatile Basic Nitrogen, Biogenic Amines and Thiobarbituric Acid Reactive Substances

Total volatile basic nitrogen (TVB-N) and thiobarbituric acid reactive substances (TBARS) were analyzed using the method previously described for pirarucu fish [12].

The quantification of biogenic amines was carried out according to the following method: during the storage period, 5 g of fish muscle (dorsal filet portion) was removed and immersed in 7 mL of 5% trichloroacetic acid (TCA) for three successive extractions, followed by filtration in HAWP membrane (0.45 mm) before analysis via high-performance liquid chromatography (HPLC) [11]. The amines were quantified in HPLC using reversed-phase column (Nova-Pak C18, 300 × 3.9 mm, 4 μm), pre-column (Nova-Pak C18, 20 × 3.9 mm, 4 µm), and fluorometric detection. All analyses were performed in triplicate.

### 2.5. Volatile Compounds and Fatty Acid Profile

The volatile compounds were extracted through simultaneous distillation and extraction (SDE) in hexane using Likens–Nickerson apparatus [13]. The gas chromatographic analyses of volatile substances and fatty acids were carried out in a GC-MS (Thermo Scientific Trace 1300 Gas Chromatographer (GC) connected to a Quadrupole Mass Spectrometer (MS), Thermo Scientific -ISQ Single, USA) with an AI 1310 autosampler and an RTX-65 TG (15 m × 0.25 mm × 0.1 µm) capillary column, using constant helium flow at 1 mL/min. The injection of extracts followed the split 1:5 mode. The injector operated at 250 °C. The MS-ISQ operated using an interface and ionization source at 280 °C, a mass range of 40–1000 Da, and electronic ionization at 70 eV. The identification of target substances was performed by comparing the mass spectra with the data available in the software WILEI2009. The concentration of free fatty acids (FFA) was established by calculating the normalization of the peak area.

### 2.6. Microbiological Analyses

Pathogenic bacteria such as coliforms at 45 °C, *Salmonella* spp., and coagulase-positive *Staphylococcus* spp. were analyzed only in the fish muscle on days 1, 11, and 18, while bacteria responsible for spoilage, such as mesophilic and psychrotrophic, were counted on days 1, 4, 8, 11, 14, and 18. All analyzes followed the methodology previously described in [14]. 

### 2.7. Statistical Analysis

All analyses were carried out in triplicate and the means were compared using analysis of variance (ANOVA). The effects were considered statistically significant by Tukey’s test when *p* ≤ 0.05. The uncertainty of the prediction of days in ice for quality index used partial least square (PLS) regression analysis, simple linear regression, and Pearson’s correlation. All regressions were calculated using the software XLSTAT for Windows version 2012.

## 3. Results

### 3.1. Developing the QIM

The QIM methodology can be based on attributes with sensory relevance in fish spoilage, which allows for verifying if the quality requirements are fulfilled [2]. The specific attributes considered relevant to assess freshness and that characterized the sensory changes in whole king weakfish over storage on ice were listed from a consensus among the referees. These attributes are shown in Table 1. The parameters assessed received up to three descriptors with demerit points ranging from 0 to 2, except for meat firmness (overall aspect), odor and condition (anal area), and shape and blood (eyes), which ranged between 0 and 1. The reason for this was the fragile muscle structure of species of the family *Sciaenidae*, as well as the rapid change in the eyelid structure that resulted in the eyes becoming flat shortly after collection.

Appearance, eyes, firmness texture and color, and mucus gills are attributes that can be used to standardize fish freshness measure (saltwater fish and freshwater fish) based on the quality index method [5,15].

The loss of freshness during storage was calculated from the sum of the scores attributed to the gradual changes in the sensory characteristics evaluated at each day of storage, which originated the QI (Figure 1B). The evolution of QI for whole king weakfish was highly correlated with the time stored in ice on lots 3 and 4 and can be expressed by the linear equations QI = 0.767x days + 8.3361 (R^2^ = 0.9661) and QI = 0.9281x days + 1.3378 (R^2^ = 0.9868), respectively. The results obtained for the lots confirmed the importance of validating the QI protocol through studies at different locations, seasons, and forms of capture. The QI linear behavior was statistically significant (*p* ≤ 0.05). The sampling period affected the highest QI values for lot 3 in relation to the quality parameters: appearance, fins, smell, and mucus from the gills, anal region, and eyeball. The results are common in fish collected after the breeding season, where more degeneration is observed [16].

Based on trained judges’ scores for lots 3 and 4, the sample QI ranged from 8 to 2 on the first day of storage, respectively. Studies indicate that the QI does not start at 0 (high freshness) due to the rapid changes that occur after capture [8]. The initial freshness for king weakfish was the best in lot 4. After 14 days of storage, lot 3 reached the maximum demerit score (QI = 21), indicating the referees had rejected the samples, which made the king weakfish improper for consumption from the sensory standpoint. However, after 18 days, the QI of lot 4 was 18, close to the maximum score in the QI protocol developed. According to the sensory results of the lots, the shelf-life of the whole hake was established at 11 days of ice storage. The results are close to those found for the fish *Scomber japonicus*, *Boops boops*, and two farmed ones (*Sparus aurata* and *Dicentrarchus labrax*) [5,6,9]. The shelf-life was longer than that of acoupa basking fish [8].

**Figure 2:**
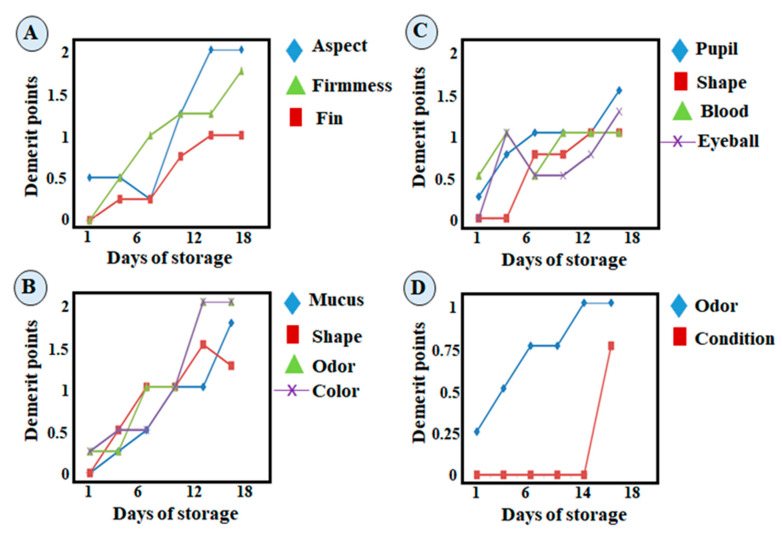
Mean demerit scores of quality attributes: overall appearance (**A**), gills (**B**), eyes (**C**), and anal area (**D**) of whole king weakfish stored in ice.

The evolution of all king weakfish quality attributes during storage in ice for lot 4 is illustrated in Figure 2A–D. In all parameters (overall appearance, gills, eyes, anal area), there was an upward trend during storage. The upward behavior observed in quality attributes was accompanied by a strong correlation between the duration of ice storage and fish spoiled. The parameters of meat firmness, fins, gill odor, and mucus, as well as anal odor, were all similar. The variables with low linearity were the anal health, eye blood flow, and eyeball, which indicates a minimal impact of these variables on the QI. The evolution of the QI over time had a better linear correlation than the individual parameters analyzed. Therefore, fish industries, suppliers, and consumers can use the QI protocol developed during the shelf-life assessment of ice-stored whole king weakfish.

### 3.2. Partial Least Squares Regression

**Figure 3:**
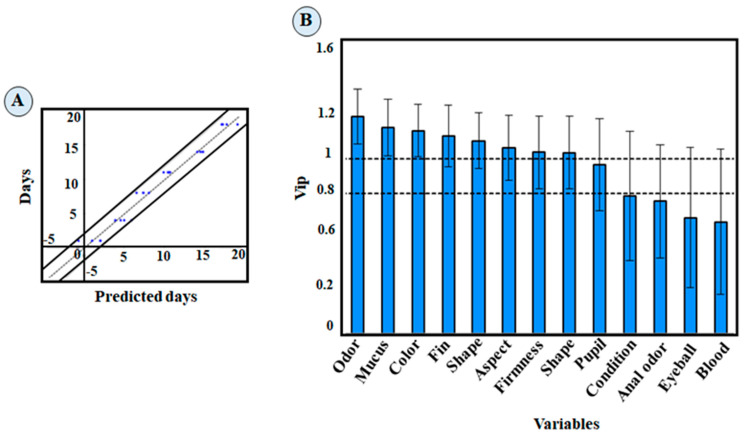
Standard error of performance (**A**) and partial least squares regression of the QIM parameters developed (**B**) for the whole king weakfish stored in ice.

The results obtained from the QI protocol were analyzed with regression to verify the efficacy in predicting whole king weakfish storage time in ice. The partial least squares regression plots and the standard error of performance (SEP) can be used to assess the precision of QI predictability (Figure 3A,B). The standard error of performance for the QI for lot 4 was 0.780. The graphs indicate that the referees’ assessments disagreed on the first days of storage (days 1, 4, and 8) with the QI scores. However, the QI values were closer in the last days, contributing to a more precise definition of the end of the shelf-life of the king weakfish.

The variables with the highest projection of importance (VIP) are those with VIP values above 1.0, that is, the most relevant variables for the statistical model used. As shown in Figure 3B, the quality attributes that stood out for the QI protocol were gills (odor, mucus, color, and shape), general appearance (meat firmness, fins, and surface appearance), and eyes.

### 3.3. Microorganism Counts Changes during the Ice-Storage of King Weakfish

The microbial growth may manifest as spoilage in fish, leading to sensory changes in the fish matrix. In order to validate the results obtained by the QIM, some authors applied microbial growth as an important parameter [1].

The results of the coliform analysis at 45 °C showed low contamination over the 18 days of storage, with values varying between 36 and 200 (CFU/g). Furthermore, *Salmonella* spp. were not found, and the *Staphylococcus* spp. count was below the limit of 10^3^ CFU/g. These microorganisms are not part of the regular microbiota of fish and, when present, may be associated with inadequate management in the production chain [17].

**Figure 4:**
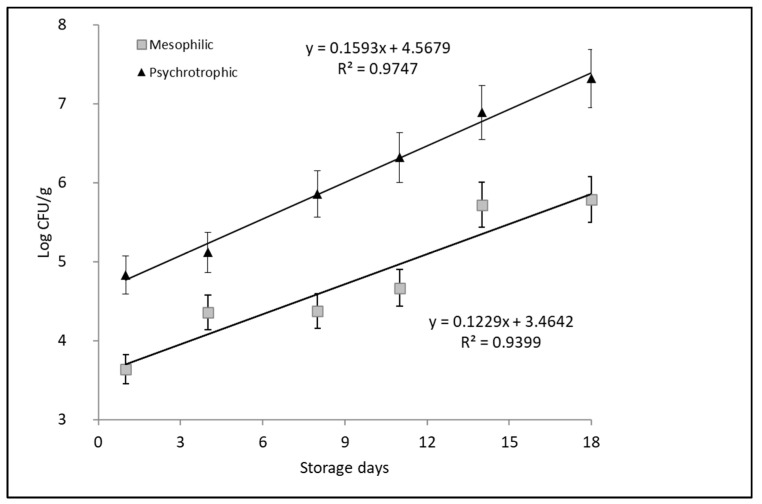
Correlations between days of ice storage and counts of psychrotrophic and mesophilic bacteria for whole king weakfish stored in ice.

The maximum detectable count of mesophilic and psychrotrophic bacteria is illustrated in Figure 4. The International Commission on Microbiological Specification for Foods recommends a maximum limit of 7 log CFU/g for standard plate counts of mesophilic and psychrotrophic aerobic bacteria in refrigerated fish [18]. The count of psychrotrophic bacteria reached 7.02 log CFU/g in the muscle of whole king weakfish after 14 of ice-storage, which is considered improper for human consumption. However, the aerobic heterotrophic mesophilic bacteria count reached only 5.7 log CFU/g after 18 days, which indicates that temperature control was effective during storage. The linear equations for the counts of mesophilic and psychotropic bacteria were expressed as Log UFC/g = 0.1229x days − 3.4642 (R^2^ = 0.9399) and Log UFC/g = 0.1593x days − 4.5679 (R^2^ = 0.9747), respectively.

These results coincide with those previously reported for spiny lobster stored in ice, which state that spoilage, especially at low temperatures, may be caused primarily by psychrotrophic bacteria [5]. The bacteria use a combination of enzymatic, oxidative processes that lead to a reduction in sensory quality through significant changes in the odor, appearance, texture, and color of the fish. Pearson’s correlation showed that psychrotrophic counts were highly correlated (0.96) with the QI score. These correlations confirm that microbiological growth is consistent with sensory evaluations. These parameters are relevant quality indicators to determine the critical day on which the sensory quality detected by the MIQ coincides with an unacceptable level of microbiological risk. These results are similar to those previously reported in *S. aurata*, *D. labrax*, *T. trachurus*, *S. colias*, and *S. quinqueradiata* fish species that showed the lowest QIM values and microbial counts on the 10th day of storage [9,19].

### 3.4. Physicochemical Changes during the Ice-Storage of King Weakfish

The moisture, proteins, and lipids were significantly different (*p* ≤ 0.05) compared to the results in the first day and end (18th day) of storage, while ash content did not significantly vary (Table 2). Over time, the moisture content of muscle increased, and the protein and lipid content decreased. This indicates changes in the quality of these components as a result of proteolytic activity occurring after fish capture and the action of endogenous bacterial enzymes. The results of the centesimal composition were similar to those found in other studies on king weakfish [20] and on acoupa weakfish [8], which belong to the same family. In a previous study on the impact of dietary lipid level on the freshness profile and others aspects in European seabass, dicentrarchus labrax, it was determined that the dietary lipid level did not have any influence on fish freshness conditions when kept on ice for 10 days at 4 °C [21].

On the other hand, it is important to highlight that centesimal composition is a characteristic used to ensure the high nutritional value of fish matrix. Nonetheless, it is a slow, destructive, and non-objective methodology to evaluate fish freshness [4]. However, the determination of the concentration of hydrogen ions, TVB-N, and TBARS can be used to assess the fish freshness and define the shelf-life of some fish product [5,12,15]. Furthermore, in the industrial sector, it does not require significant economic efforts to be implemented or maintained.

At the beginning of the shelf-life study (1 days), the pH value agrees with the value of 6.8 found by another author of a study about fish conserved on crushed ice at 0 °C [15]. The pH differed significantly (*p* ≤ 0.05) throughout the storage period, except between the eighth and eleventh days, reaching a value of 7.48 on the last day of storage. pH values tend to increase with storage due to the accumulation of volatile nitrogen compounds, such as peptides, amines, and ammonia, formed from autolytic and bacterial activities [1,7]. This can be observed for king weakfish, which had a significant increase in pH on the 14th day, along with microbiological and sensory changes, and is, therefore, an essential parameter for evaluating fish freshness, unlike what was observed in the studies for tilapia (*Oreochromis niloticus*) [1] and for mackerel (*Scomber japonicus*) [5], where they did not observe a significant relationship between pH and sensory changes. Regarding the firmness of fish muscle, no significant differences (*p* ≥ 0.05) were observed during the 18 days of ice storage.

Table 3 shows the profile of fatty acids, which are quality markers in fish products. Palmitic acid is normally detected at high amounts (10.6 to 23.7%) in several marine species [11,22]. It is possible that the chain of the stearic acid (C18) is elongated, making it possible to synthesize other unsaturated fatty acids important for food [23].

There was also a large proportion of C18 and C20 fatty acids divided into monounsaturated and polyunsaturated fatty acids. There was a reduction in Oleic and Arachidonic acids during storage, indicating that the deterioration was related to changes in sensory characteristics caused by oxidative processes. The volatile organic substances, such as benzene, toluene, xylene, and alkanes, are petroleum hydrocarbons found in marine and coastal ecosystems probably derived from illegal sewage of oil tankers and industrial effluents among other sources. The presence of such volatile substances in the king weakfish indicates that the specimens accumulated toxic petroleum compounds, usually present in the sediment of coastal zones inhabited by this species. The proximity of sample collection to the Pará River, an area of intense traffic of ships and boats, might also favor sporadic spills of oil and other chemicals [24].

These hydrocarbons can damage organisms by causing structural and functional damages. This is because these compounds are lipophilic, so they can be absorbed into animal tissues. The increased presence of volatile substances in the king weakfish is probably caused by the release of these compounds during deterioration [21]. Amine determination in some fish can be used as a good toxicity marker, since some amines may potentialize histamine [25]. During 14 days of king weakfish ice storage, putrescine was the amine with the highest levels. Spermidine and cadaverine were found only on the fourth and fourteenth days. During the storage period, putrescine, cadaverine, and spermidine tend to increase because of the degradation of arginine, lysine, and spermidine [21].

According to previous studies, values below 10 mg/kg for putrescine indicate high-quality fresh fish, while values above 20 mg indicate low quality [11,26]. In comparison with the data found in the present research, king weakfish muscle marked a loss of quality from the eighth day of storage. However, the rejection threshold established by the QI (sensory method) occurred after the 11th day. Despite the high value of putrescine, the referees were unable to detect it through odor. Cadaverine was detected on the 4th day of storage at a level above the limit of 10.8 mg/kg for red mullet and 13.3 mg/kg for gold goat fish after 11 days of ice storage [27]. The presence of spermidine had no harmful effects on the fish quality, since this amine is naturally present in animal tissues.

**Figure 5:**
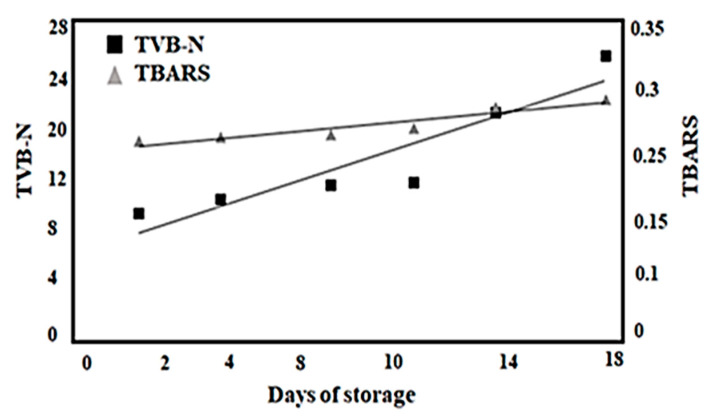
Correlations between days of ice storage the chemical changes in TVB-N and TBARS for whole king weakfish stored in ice.

In what concerns the TVB-N and TBARS parameters, the results for king weakfish are shown in Figure 5. These physicochemical parameters had a good linear correlation during the shelf-life assay. The linear equations were expressed as follows: TBARS (mgMDA/kg) = 0.0034x days + 0.2253 (R^2^ = 0.8849) and N-BVT (mgN/100 g) = 0.9577x days − 8.0379 (R^2^ = 0.8488). The TBARS values found in the present study are considered low because king weakfish has a low amount of muscle lipid (between 1 and 2 g/100 g). However, lipid oxidation progressively increased during storage, thus favoring the rise of malondialdehyde from 0.235 to 0.291 mg MDA/kg after 18 days of storage. This content significantly differed (*p* ≤ 0.05) after the 11th day of storage. Previously studied in *Sardine pilchardus* and *Boops boops*, the suggested limits are 5 to 8 mg MDA/kg for the sensory acceptance of fish [6]. Although this parameter may be strongly related to fish degradation due to high levels of unsaturated fatty acids [4], in this study, its increase during storage was non-significant and the king weakfish samples remained well below this limit throughout storage.

The TVB-N values tended to increase, ranging from 11.06 mg N/100 g on the first day to 28.4 mg N/100 g by the end of storage at 18 days; this can be associated with the protein degradation found in this study that was aforementioned and previously described [4]. There was no significant difference (*p* ≥ 0.05) on the first day of storage, and only after the fourteenth. However, the king weakfish samples remained within the limit of 30 mg N/100 g and of 35 mg N/100 g [4,10]. These results match those observed for shelf-life of acoupa weakfish and bogue [6,28]. TVB-N is not considered a good indicator of freshness and is not very reliable for several species since it is not linearly correlated with spoilage during ice-storage [10,16]. According to some studies, the leaching of these compounds is an important factor in lowering the efficiency of TVB-N results in determining freshness [28].

## 4. Conclusions

The quality index method (QIM) developed for whole hake (*Macrodon ancylodon*) represents a reliable tool for assessing the freshness of this fish and can be used in an industrial environment if its specific storage conditions are adopted in ice at a ratio of 1:1 (m/m) with daily renewal, which will maintain the freshness and quality of the fish for 11 days with commercial stability. Also, considering the analysis methods used in this research, which indicated a loss of freshness that occurs gradually over time, this was proven by analyzing greater correlations between microbiological and sensory rejection. This showed that the sensory evaluation did not encounter limitations in its development and validation, either by the team composed of workers from the fish factory or by the application of the QIM protocol in the different seasonality of the batches. This demonstrates good prospects due to companies’ ease of implementing the sensory protocol and more simplified personnel training.

## Figures and Tables

**Table 1:** QIM sensory analysis protocol developed for king weakfish stored in ice.

Quality Attributes	Parameters	Description of Characteristics	Demerit Points
OVERALL ASPECT	Surface aspect	Intense glisten, characteristic pigmentation	0
		Glistening, more opaque colors	1
		Little glisten, depigmentation	2
	Meat firmness	Firm, little elastic	0
		Tender, signs of pressure	1
	Fin	Very elastic	0
		Little elastic	1
		No elasticity	2
GILLS	Color	Bright red to dark red	0
		Less-bright red	1
		Less-bright red to pink	2
	Mucus	Little mucus, clear	0
		Consistent mucus, opaque	1
		Much mucus, opaque	2
	Odor	Algae (slightly wet sand)	0
		Neutral, less intense algae	1
		Slightly rancid	2
	Shape	Whole	0
		Slightly misshapen	1
		Misshapen	2
EYES	Eyeball	Clear (transparent)	0
		Slightly opaque	1
		Milky, opaque	2
	Pupil	Bluish-black, well defined	0
		Foggy and defined	1
		Foggy and undefined	2
	Shape	Squashed (flat)	0
		Depressed (concave)	1
	Blood	Absent	0
		Slightly bloody	1
ANAL AREA	Odor	Fresh (algae)	0
		Slightly rancid	1
	Condition	Closed	0
		Open	1
**Quality Index (QI)**			0–21

**Table 2:** Results of the physicochemical analyses of king weakfish stored in ice.

Analyses	Ice-Storage Days					
	1	4	8	11	14	18
Moisture (g/100 g)	80.7 ± 0.41	-	-	-	-	81.38 ± 0.23
Proteins (g/100 g)	17.68 ± 0.18	-	-	-	-	16.65 ± 0.30
Ashes (g/100 g)	1.00 ± 0.20	-	-	-	-	0.97 ± 0.15
Lipids (g/100 g)	1.97 ± 0.37	-	-	-	-	1.00 ± 0.10
pH	6.80 ± 0.20	6.94 ± 0.30	7.06 ± 0.20	7.11 ±0.15	7.35 ± 0.34	7.48 ± 0.23
Texture (N)	11.73 ± 0.33	11.88 ± 0.34	11.35 ± 0.40	11.38 ± 0.40	11.14 ± 0.45	11.01 ± 0.48

**Table 3:** Profile of fatty acids, volatile organic substances, and biogenic amines in ice-stored king weakfish average samples.

Parameters	Ice-Storage Days					
Fatty Acids (g/100 g)	**1**	**4**	**8**	**11**	**14**	**18**
Myristic acid (C14:0)	1.33	0.78	0.58	1.45	0.88	1.49
Palmitic acid (C16:0)	1.37	9.02	8.51	10.82	11.02	12.06
Stearic Acid (C18:0)	nd	3.26	3.37	3.28	nd	3.64
Palmitoleic acid (C16:1 n-7)	6.30	10.89	8.80	7.22	6.42	6.38
Oleic acid (C18:1 ω-9)	26.97	23.79	22.10	16.48	9.48	14.90
Arachidonic acid (C20:4 ω-6)	9.19	3.12	8.96	1.35	1.09	2.40
Volatile organic substances (%)						
Toluene	9.92	-	14.77	-	-	30.68
Ethylbenzene	3.03	-	4.84	-	-	8.87
Xylene	19.90	-	34.00	-	-	60.43
Total alkane	67.14	-	46.40	-	-	-
Biogenic amines (mg/kg)						
Putrescine	3.28	13.74	24.41	65.84	409.97	-
Cadaverine	-	15.22	-	-	55.54	-
Spermidine	-	0.69	-	-	1.71	-

Standard deviation for fatty acids was ±0.01; volatile organic substances were between 0.01 ± 0.03, and biogenic amines were <0.01.

## Data Availability

The original contributions presented in the study are included in the article, further inquiries can be directed to the corresponding author.
